# Conserved, yet disruption-prone, gut microbiomes in neotropical bumblebees

**DOI:** 10.1128/msphere.00139-23

**Published:** 2023-10-19

**Authors:** Nickole Villabona, Nancy Moran, Tobin Hammer, Alejandro Reyes

**Affiliations:** 1 Research Group on Computational Biology and Microbial Ecology, Max Planck Tandem Group in Computational Biology, Department of Biological Sciences, Universidad de Los Andes, Bogotá, Colombia; 2 Department of Integrative Biology, University of Texas at Austin, Austin, Texas, USA; 3 Department of Ecology and Evolutionary Biology, University of California, Irvine, California, USA; 4 The Edison Family Center for Genome Sciences and Systems Biology, Washington University School of Medicine, St. Louis, Missouri, USA; University of Wisconsin-Madison, Madison, Wisconsin, USA

**Keywords:** *Bombus*, symbiosis, microbiota, bacteria, pollinators, Neotropics, bees

## Abstract

Bumblebees are important pollinators in natural ecosystems and agriculture, but many species are declining. Temperate-zone bumblebees have host-specific and beneficial gut microbiomes, which may have a role in mediating the effects of stressors. However, there is almost no published information on the gut microbiomes of tropical bumblebees. As temperate and tropical bumblebees encounter different floral resources and environmental conditions, their microbiomes could differ. Here, we characterized the gut microbiomes of four neotropical *Bombus* species and, for comparison, co-occurring solitary bees (genus *Thygater*). We collected wild-foraging bees from multiple sites in central Colombia and used 16S rRNA gene sequencing to characterize their gut microbiomes. DNA barcoding and morphology were used to identify bumblebee species. We found that the microbiomes of neotropical bumblebees cluster with those of closely related temperate-zone species, in agreement with a model of bumblebee-symbiont codiversification. There was no evidence of geographic differences in microbiome composition between neotropical and temperate-zone bumblebees. These results suggest that the microbiome was conserved during bumblebee dispersal from North America, despite major shifts in ecology and life history. As previously observed in temperate-zone species, some neotropical bumblebees have highly disrupted microbiomes, in which conserved gut bacterial symbionts are replaced by environmental microbes. In these individuals, the gut microbial profile is more like that of solitary bees than of conspecifics. The gut parasites *Nosema* and *Crithidia* are also prevalent and associated with microbiome disruption. Our findings provide insights into the biogeography of bee microbiomes and a foundation for studying bee-microbe-stressor interactions in the neotropics.

**IMPORTANCE:**

Social bees are an important model for the ecology and evolution of gut microbiomes. These bees harbor ancient, specific, and beneficial gut microbiomes and are crucial pollinators. However, most of the research has concentrated on managed honeybees and bumblebees in the temperate zone. Here we used 16S rRNA gene sequencing to characterize gut microbiomes in wild neotropical bumblebee communities from Colombia. We also analyzed drivers of microbiome structure across our data and previously published data from temperate bumblebees. Our results show that lineages of neotropical bumblebees not only retained their ancient gut bacterial symbionts during dispersal from North America but also are prone to major disruption, a shift that is strongly associated with parasite infection. Finally, we also found that microbiomes are much more strongly structured by host phylogeny than by geography, despite the very different environmental conditions and plant communities in the two regions.

## INTRODUCTION

The symbiosis between social corbiculate bees and their specialized gut bacteria is ancient (1). Gut microbiomes influence bee health by contributing to digestion and protection from parasites and pathogens (2). Disturbance to these symbionts may therefore decrease the ability of bees to obtain nutrients or ward off disease.

Many species of bumblebees in the temperate zone have relatively well-characterized gut microbiomes, consisting of ~4–5 core bacterial symbiont groups that are highly conserved (1, 3–6). Approximately 42 species of bumblebees (Apidae: *Bombus*) live in the neotropics, but there is no published information on their gut microbiomes, aside from one study including two individuals (7). This knowledge gap limits our understanding of how microbiomes may mediate bumblebee responses to environmental change in tropical ecosystems.

Climate change is expected to strongly impact low-latitude and high-elevation biodiversity, forcing animals and plants to either adapt, disperse, or go extinct (8). Temperate-zone bumblebees are already shifting or contracting their ranges in response to global warming (9, 10). The data on responses of neotropical bumblebees to thermal stress are very limited, and these responses might be modified by the gut microbiome (11). Although core gut bacterial symbionts of temperate-zone bumblebees appear to be quite robust to high temperatures (12), we do not know if these bacteria are generally conserved in tropical bumblebees.

Comparing neotropical and temperate bumblebee gut microbiomes provides an opportunity to examine the biogeography of host-microbe symbioses. For macroorganisms, the latitudinal diversity gradient is a well-documented and common biogeographic pattern, with low latitudes typically harboring high diversity (13). For host-associated microbiomes, a range of relationships between latitude and microbiome diversity have been reported (14–18), indicating a lack of a general pattern. Within social bees, current data also suggest a mixture of patterns. At a narrow phylogenetic scale (within *Apis* species) and geographic extent (10 degrees of latitude), honey bee gut microbiomes were more diverse at lower latitudes (19). On the other hand, the diversity of gut microbiomes in exclusively tropical Meliponini (stingless bees) is generally similar to that in temperate bumblebees (2). Large-scale comparisons, focused on individual bee clades such as bumblebees, have not yet been reported.

Bumblebees are thought to have originated in montane environments of Central Asia and are most abundant and diverse at high latitudes and elevations (20, 21). Through two independent waves of dispersal, bumblebees colonized South America from North America relatively recently (~3.5–11 mya). As these colonists adapted to environmental conditions in the neotropics, their gut microbiomes may have changed. For example, unlike higher-latitude species, neotropical bumblebees do not undergo diapause (a state roughly similar to hibernation), and some species can have extremely large colonies (22, 23). These life history traits might allow a greater diversity of gut microbes to be maintained and transmitted between generations (6). Neotropical bumblebees also inhabit unique habitats (e.g., lowland rainforest and high-elevation *páramo*s) and forage from distinct pollen and nectar sources, potentially altering selective pressures on the microbiome.

In this study, we examined the composition of the gut microbiome from five species of bumblebees and one solitary bee species (Apidae: *Thygater aethiops*), collected in Colombia. *Thygater aethiops* bees were collected to assess whether the gut symbionts present in bumblebees might be shared with the co-occurring members of the wider bee community. Both of the hypothesized waves of bumblebee dispersal into the neotropics are represented in our samples: *Bombus hortulanus*, *Bombus rubicundus*, and *Bombus robustus* belong to the first wave (~11 mya); *B. atratus* belongs to the second wave (~3.5 mya) (21). We analyzed the neotropical bumblebee gut microbiomes through 16S rRNA gene sequencing and compared them to previously surveyed microbiomes of North American bumblebees. Our work provides comprehensive data on core gut symbionts and putative parasites of neotropical bumblebees.

## MATERIALS AND METHODS

### Study sites and sample collection

**Fig 1:**
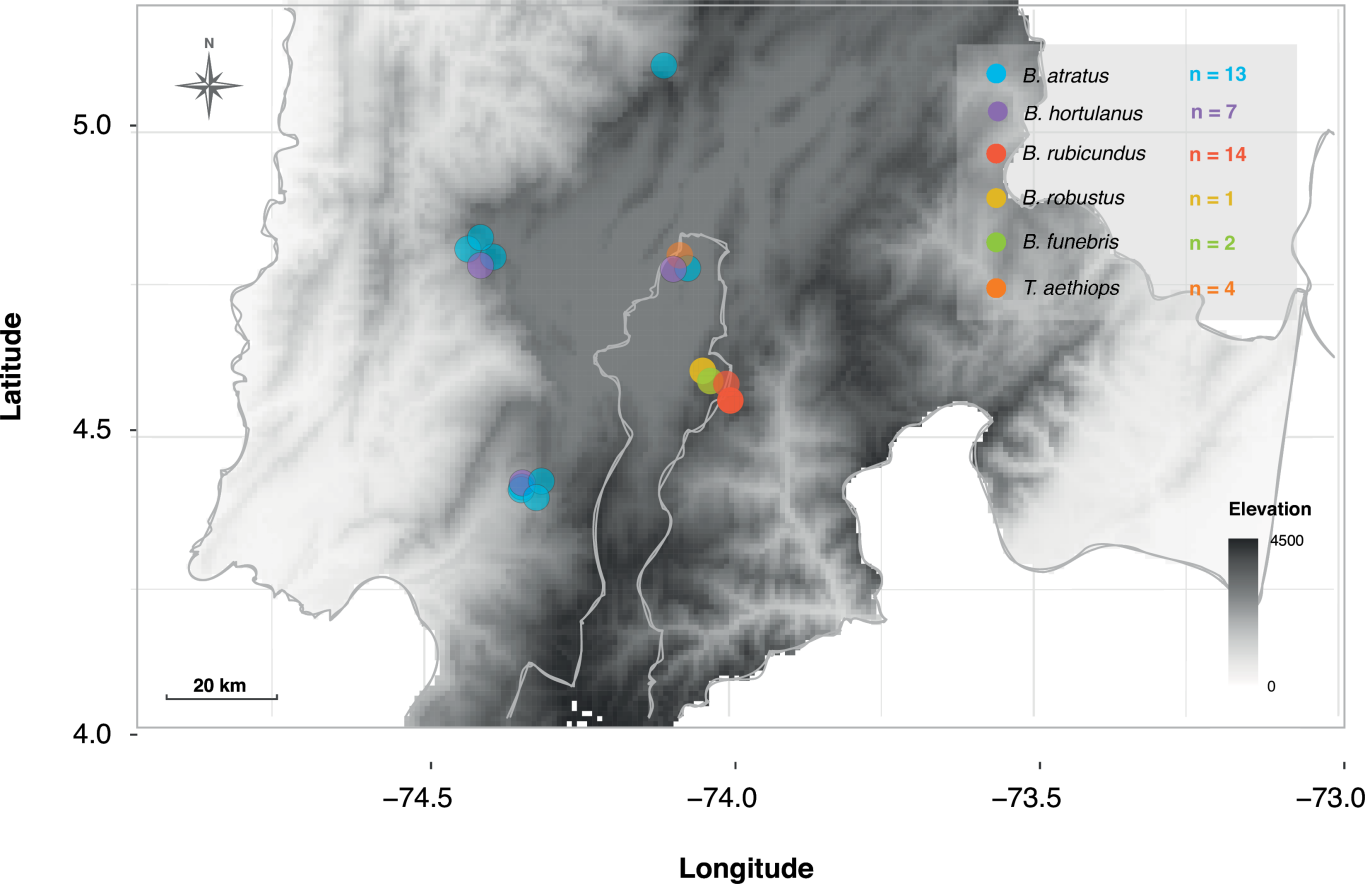
Map of Cundinamarca, a department of central Colombia depicting sampling sites. Each species is represented by a different color, and elevation is shown as a black-white gradient.

We sampled from three habitats in Cundinamarca, Colombia: (i) 1,500–2,600 masl (meters above sea level), corresponding to highland prairies; (ii) 2,600–3,100 masl, sub-páramo; and (iii) 3,100–3,800 masl, páramo. Each habitat has a distinct climate and plant community. From January to May 2019, we sampled eight locations that were separated by a mean of 15 km to minimize the probability of collecting bumblebees from the same colony (Fig. 1). Bees were captured with nets in the field while foraging. Most of them were workers (*N* = 34), but some males (*N* = 2) were collected as well. The specimens were taken to the laboratory, and their whole guts were aseptically dissected and preserved in a custom buffer for RNA preservation at −80°C as described in a previous protocol (24). Bee specimens were preserved in 70% ethanol for morphological identification and DNA barcoding. Since several of the collected bumblebees did not have a reference sequence of the mitochondrial cytochrome oxidase I (COI) gene in public databases, we carried out morphological identification using a key to Colombian *Bombus* species (25) to assign the correct species to each sample and validated using COI gene sequences. All the corresponding collection (#IDB0359) and export permits (#440076030026092019) were processed through the Universidad de Los Andes.

### Sequencing amplicons of 16S rRNA and COI genes

Guts were manually homogenized with a sterile pestle in 500 µL of CTAB (cetyltrimethylammonium bromide). DNA was extracted using a protocol described in reference 26. Three blanks were included in the extractions. The final DNA extracts were quantified using the Qubit dsDNA broad-range assay (Invitrogen) and stored at −20°C until sequencing.

16S rRNA gene amplicon library preparation and sequencing were performed for a total of 36 samples and three blank negative controls as a service at the Center for Genome Sciences and Systems Biology, Washington University School of Medicine. For the amplification and sequencing of the V4 hypervariable region of the 16S rRNA gene, the primers 515F and 806R were used following a previously described protocol (27). Sequencing was performed on an Illumina MiSeq platform with 2 × 250-bp paired-end reads. Raw reads are available in the ENA database (accession number: PRJEB42751).

The bee barcoding procedure was performed by the Canadian Centre for DNA Barcoding (CCDB; Guelph, Ontario, Canada; http://ccdb.ca/). Bee tissue (either a midleg or gut homogenate) was sent to the CCDB, and DNA extraction, amplification of the COI barcode (primers Lep-F1 [5′-ATT CAA CCA ATC ATA AAG ATA T-3′] and Lep-R1 [5′-TAA ACT TCT GGA TGT CCA AAA A-3′]) and sequencing protocols were performed as previously described (28). Sequences were submitted to the Barcode of Life Database (http://www.boldsystems.org) for taxonomic assignment. All COI sequences used in this study are available in the BOLD database; sequence IDs can be found in Table S1.

### Analysis of COI data

A phylogenetic reconstruction of the genus *Bombus* was performed to connect the morphological and molecular classifications. Sequences were aligned with MAFFT (28), and the phylogeny was constructed with the PhyML tool v3.3_1 (29) and GTR substitution model (30).

### Processing and analysis of 16S rRNA amplicon data

Trimmomatic v0.38 software (31) was used to filter low-quality reads using a sliding window score of 4 and a minimum Phred score of 20. A headcrop of 15 bp and a final minimum length cutoff of 180 bp was performed for all samples. Trimmed sequences were processed with QIIME 2 v2019.10 (32). Using DADA2 (33), we proceeded with denoising, truncating to a length of 230 for each read, chimeric sequence filtering (isBimeraDenovo function as default for chimera detection in DADA2), and the construction of the amplicon sequence variant (ASV) table. The feature tables were filtered using QIIME 2 feature-table filter-features (--p-min-frequency 10 and --p-min-samples 2). The sequencing resulted in a total of 1,958,207 reads, 252 ASVs, and a mean read count per sample of 46,623 before filtering. After filtering, we ended with a total of 1,920,659 reads and 101 ASVs. The ASV table can be found in Table S2.

Taxonomy was assigned to ASVs with the q2‐feature‐classifier (34) classify‐sklearn using a naïve Bayes classifier trained with SILVA v132 taxonomic reference database (34, 35). Some microbial eukaryotes were found. As they are not in the SILVA reference database, the taxonomic identifications for these ASVs were conducted using BLAST. One ASV corresponds to the parasitic microsporidian *Nosema* sp., and one ASV corresponds to the parasitic trypanosomatid *Crithidia bombi*. Even though these parasites are not bacteria, their small subunit rRNA genes were amplified using our 16S rRNA gene primers.

Alpha diversity (Shannon index) and beta diversity metrics (Bray-Curtis dissimilarity) were calculated using the “diversity” plugin in QIIME 2 with a sampling depth of 2,000 sequences per sample. As *Bombus funebris* specimens did not pass this filtering, they were excluded from these analyses. The three DNA extraction blanks had fewer than 250 reads and were also excluded. Because of the low read counts, it was not possible to rigorously identify potential contaminants. The core bumblebee taxa we report are host-restricted symbionts, but we acknowledge that some of the noncore bacteria present in *Thygater* or bumblebees with disrupted microbiomes could be reagent contaminants.

Alpha diversity differences among neotropical bumblebee species were analyzed with ANOVA. ADONIS, as implemented in the vegan R package (36), was used to test if microbial community composition differed among host species. The “betadisper” function, also in vegan (36), was used to test whether intraspecific variability in community composition (dispersion) varied between species. A heatmap of the 30 most abundant shared ASVs was generated and visualized in R (V4.0.5) using ggplot. We used ANCOM-BC (37) to find ASVs that differed in relative abundance between *Bombus* species. *B. robustus* was excluded from this analysis because it had fewer than three samples. We also excluded the solitary bee *Thygater* to focus on bumblebee-associated taxa. Finally, we tested an association between bumblebee microbiome disruption (disrupted versus core-dominated gut microbiomes) and the presence or absence of parasites using Fisher’s exact test. Disrupted microbiomes were classified as those with <30% total relative abundance of corbiculate core bacterial taxa.

### Processing and meta-analysis of published 16S rRNA amplicon data

We compared gut microbiome composition between the bumblebees used in this study and temperate-zone bumblebees. A search was carried out for 16S rRNA gene amplicon studies of the gut microbiomes of wild bumblebees, listed in reference 6. Only two studies (1, 38), both involving bees collected in the United States, used amplicons for the same region and length and were sequenced by the same technology as in our study. Since the Powell et al. (26) study used single-end sequencing, we reanalyzed our data and the (1) data using only the forward reads. Alpha diversity differences between temperate and tropical bumblebee microbiomes were evaluated by a linear mixed-effects model, with geographical region (temperate vs neotropical) as a fixed effect and host species as a random effect (model formula: Shannon ~ region, random = ~ 1|species). Beta-diversity patterns among bumblebees were evaluated by ADONIS (model formula: distance_matrix ~ region × subgenus) and by betadisper as described above.

## RESULTS

**Fig 2:**
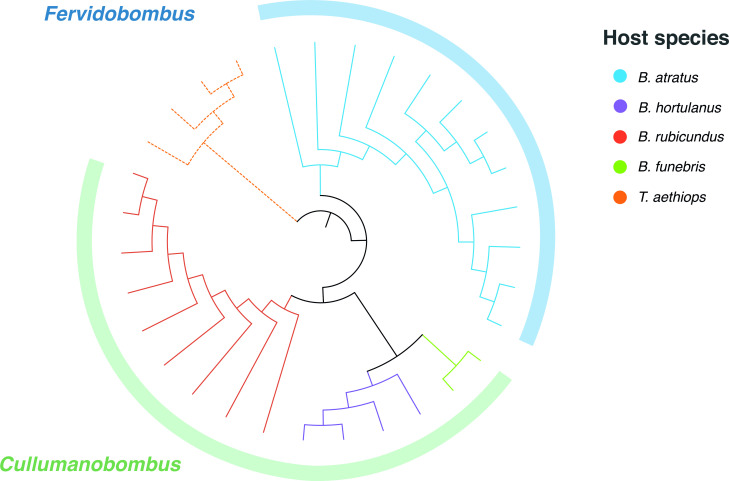
COI-based phylogenetic reconstruction of the bee species sampled in this study with the PhyML tool. The outer circle corresponds to the bumblebee subgenera.

Our COI-based phylogeny is largely consistent with published phylogenies using other markers and with taxonomic classifications of bumblebee subgenera (Fig. 2). For example, *Bombus atratus*, which belongs to the subgenus *Fervidobombus*, forms a distinct clade from the other sampled *Bombus* species, which belong to subgenus *Cullumanobombus* (21).

**Fig 3:**
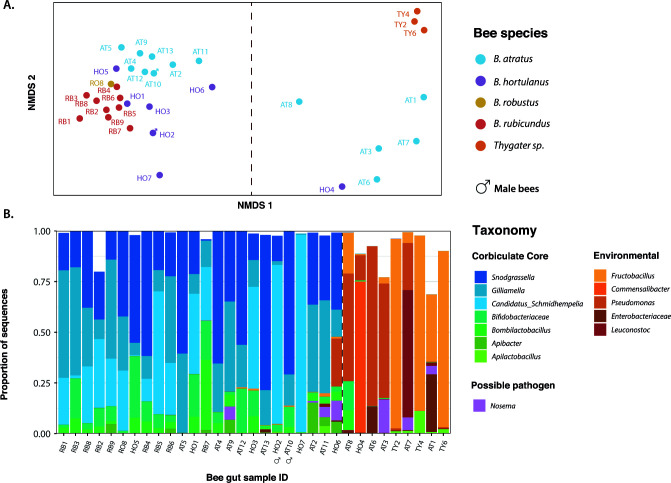
(A) NMDS visualization of gut microbiome compositional similarity. Each dot corresponds to an individual bee and the color of the bee species. The male icon represents the two males we included in the analysis. (**B) **Gut microbiome composition of bumblebees at the genus level. Each bar corresponds to a bee sample, and the relative abundance of each genus is represented by different colors. Blue-green genera are the core gut symbionts of corbiculate bees, and yellow-red genera are non-core gut microbes. The dashed vertical line divides disrupted (right) from core-dominated bumblebees (left). The samples are sorted by their coordinate on the first component of the NMDS.

The dominant core gut bacterial groups of bumblebees known from temperate species (*Gilliamella*, *Snodgrassella*, *Bifidobacteriaceae*, *Schmidhempelia*, *Bombilactobacillus*, and *Apilactobacillus*) make up over 90% of the gut microbiome in 24 of the 33 neotropical bumblebee individuals we sampled (Fig. 3B). The two males were included in these analyses along with the female workers since they did not appear to differ in composition (Fig. 3). Among bumblebees with core-dominated microbiomes, different host species have distinct microbiome profiles (Fig. 3A) (ADONIS, *R*²=0.46, *F* = 5.67, *P* < 0.05), despite overlapping genus-level composition (Fig. 3B) and similar alpha diversity (ANOVA, *F* = 1.442, *P* = 0.246). Dispersion does not significantly vary among host species (betadisper test, F = 2.63, *P* = 0.077), suggesting that the ADONIS results are largely driven by species differences in microbiome composition as opposed to dispersion.

**Fig 4:**
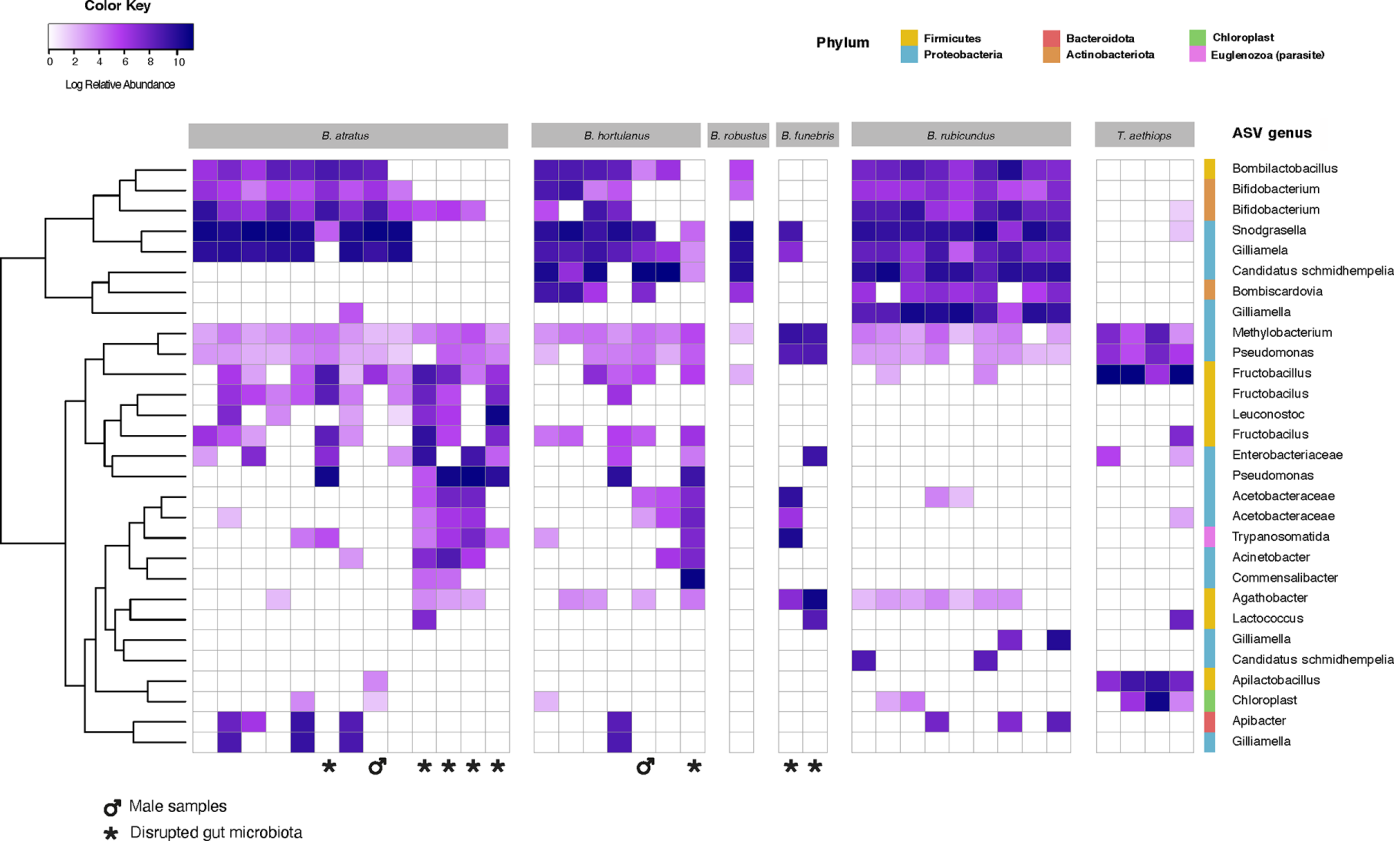
Heat-map showing relative abundances of gut microbes present in each sequenced bumblebee. The rows represent the ASVs, and the columns represent the bee individuals. ASVs with a family-level classification did not have a genus-level classification in SILVA. The symbol “*” represents a disrupted sample, and the male icon represents the two males we included in the analysis.

The presence of host-species-specific amplicon sequence variants (ASVs) within shared bacterial genera contributes to interspecific differences in microbiome composition. For example, *B. rubicundus* has a highly abundant *Gilliamella* ASV that is almost absent from other co-occurring *Bombus* species (Fig. 4). The ANCOM-BC analysis showed that 41 ASVs significantly differ in relative abundance among host species, belonging to corbiculate core taxa including *Schmidhempelia*, *Bifidobacterium*, *Bombiscardovia*, *Gilliamella*, and *Lactobacillus bombicola* (Fig. S1; Table S3).

Gut microbiomes of the solitary bee *Thygater aethiops* are composed of apparently environmental bacteria (Fig. 3B and 4): *Fructobacillus*, often associated with flowers and other plant material (36); *Pseudomonas*, common in many environments including guts of solitary bees (37); and *Apilactobacillus*, common in social corbiculate and solitary bees and in bee-associated habitats such as hive material and floral nectar (37). *Thygater* samples also contain a high proportion of chloroplast sequences (Fig. 4), suggesting relatively low bacterial abundance.

Although most sampled bumblebees harbored the conserved core symbionts, some individuals of *B. atratus* and *B. hortulanus* had aberrant gut microbiome composition. In these individuals, putative environmental bacteria dominated, and core symbionts were depleted or almost absent (Fig. 3 and 4). These bumblebees’ microbiomes were more like microbiomes of the solitary bee *Thygater sp.* than microbiomes of conspecifics (Fig. 3A). Notably, the bacteria that replace the core symbionts vary among individuals (Fig. 3B). These bacteria include *Fructobacillus* and *Leuconostoc* (*Leuconostoceae*), *Pseudomonas* (*Pseudomonadaceae*), and various *Enterobacteriaceae*, and *Acetobacteraceae* (Fig. 3B).

**Fig 5:**
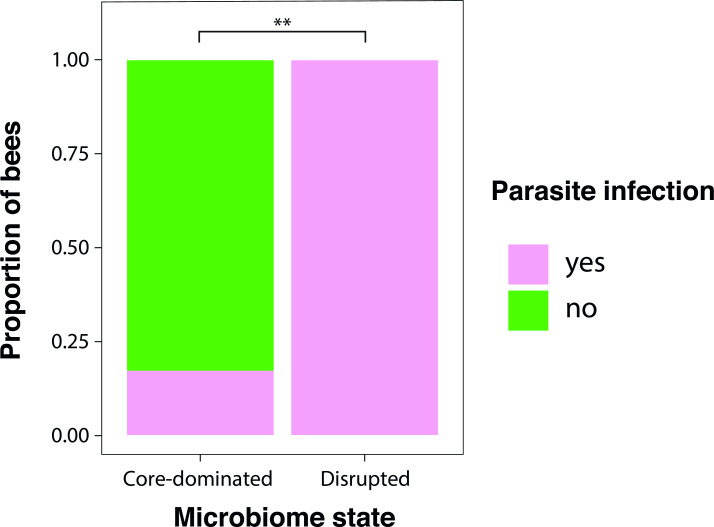
Proportion of neotropical bumblebees infected by parasitic microbial eukaryotes (*Crithidia* and/or *Nosema*). *N* = 24 bees with core-dominated microbiomes. *N* = 6 bees with disrupted microbiomes (**Fisher’s exact test, *P* = 0.0035).

Our 16S rRNA gene sequencing approach detected eukaryotic parasites (see Materials and Methods). Hence, we tested for an association between parasite infection and disruption of the gut bacterial community. Only 16% (*N* = 24) of bumblebees with core-dominated gut microbiomes harbored parasite sequences (*Nosema* and/or *Crithidia*), while 100% (*N* = 6) of the bumblebees with disrupted microbiomes harbored parasites (Fisher’s exact test, *P* = 0.0035) (Fig. 5).

**Fig 6:**
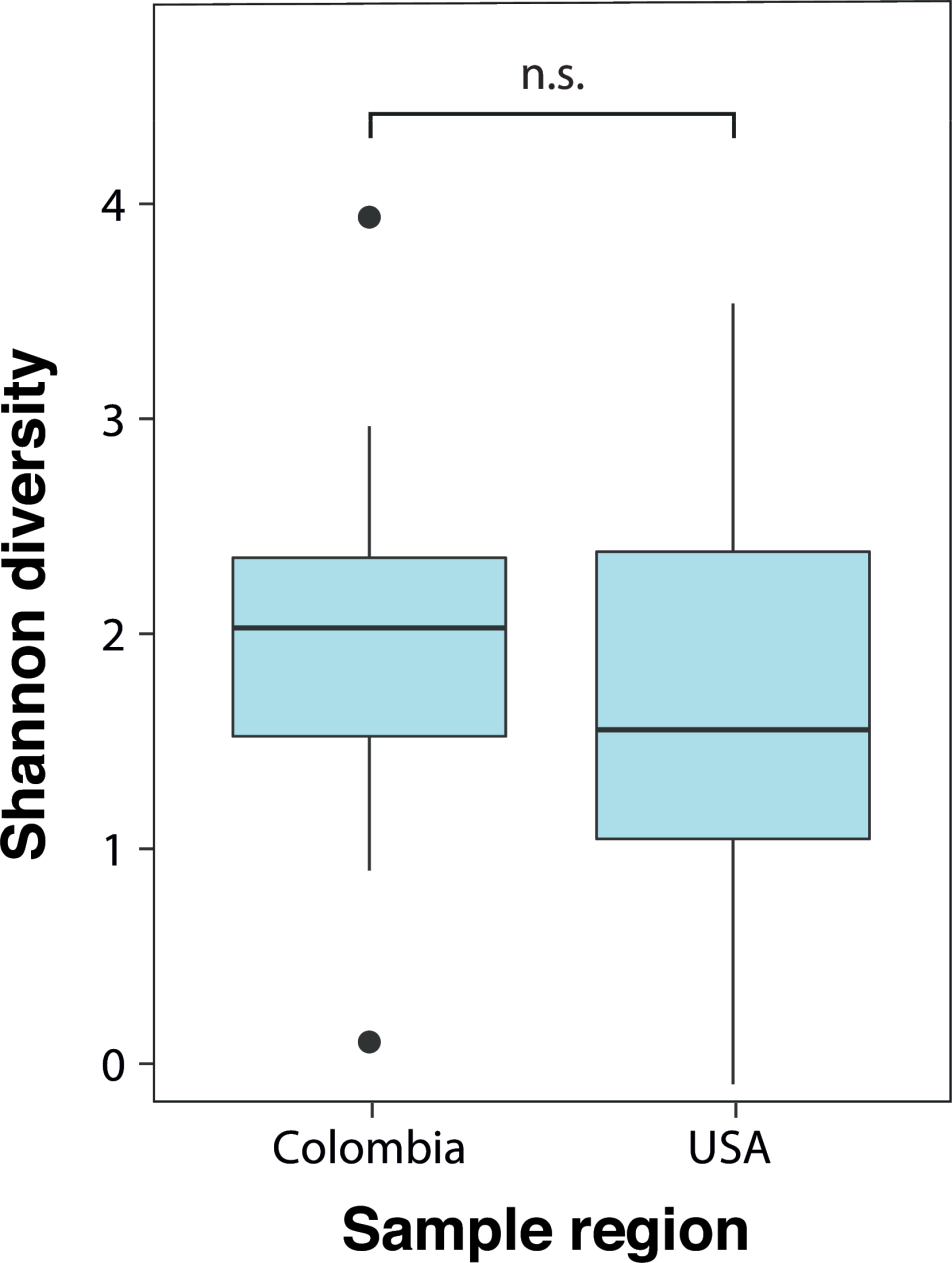
Alpha diversity (Shannon index) of bumblebee gut microbiomes in tropical and temperate regions. *N* = 81 bees from the temperate zone. *N* = 30 bees from the neotropics (NS, linear mixed-effects model, *t* = −1.059, *P* = 0.30

**Fig 7:**
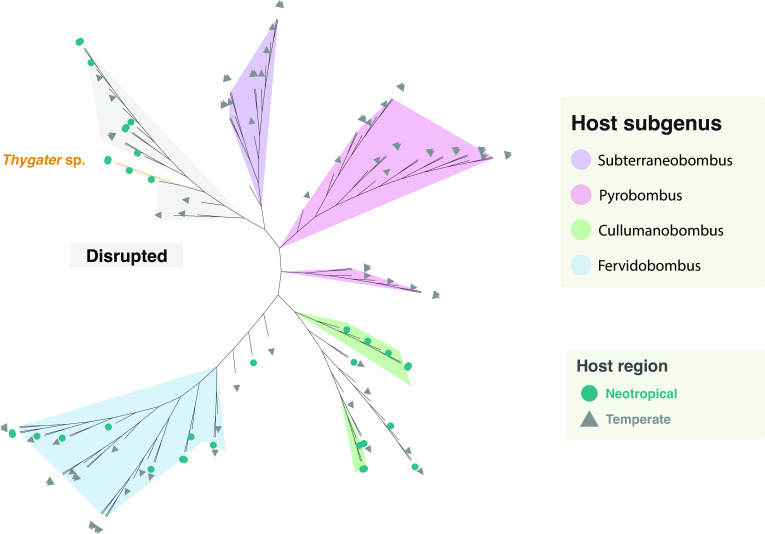
Dendrogram of microbiome compositional differences among bumblebees (Bray-Curtis dissimilarities), comparing neotropical and temperate species. The branches are shaded when they correspond to clusters belonging to the same subgenus or a cluster with disrupted microbiomes.

To quantitatively compare bumblebee microbiomes between temperate and neotropical regions, we reanalyzed 16S rRNA gene sequence data from references 1, 38 using the same methodology. Microbiome alpha diversity does not significantly differ between temperate and neotropical bumblebees (linear mixed-effects model, *t* = −1.059, *P* = 0.307) (Fig. 6). Similarly, bumblebee gut microbiome composition is not structured by latitude. Instead, microbiomes cluster primarily by host phylogenetic relationships, with neotropical microbiomes intermixed with temperate microbiomes in either disrupted or host-subgenus-specific clusters (Fig. 7). ADONIS analysis confirmed a strong association between microbiome composition and host subgenus (*R*²= 0.23, *F* = 5.67, *P* < 0.05). This effect is partly driven by differences in dispersion (betadisper test, *F* = 6.68, *P* < 0.05), but distinct subgenus-specific clusters are also clearly evident (Fig. 7). The effect of latitude is statistically significant but weak (*R*²= 0.049, *F* = 7.25, *P* < 0.05).

## DISCUSSION

We found that neotropical bumblebees harbor gut microbiomes with similar composition as temperate-zone bumblebee species, indicating that they retained their core gut symbiont lineages during dispersal from North America. This conservation is present despite environmental, ecological, and life history differences between temperate and neotropical *Bombus*. The five neotropical bumblebee species we studied share the same core symbiont taxa but also present differences in community composition. These differences appear to be partly driven by host-specific ASVs occurring alongside more widely distributed ASVs. Core bumblebee taxa such as *Snodgrassella* and *Gilliamella* have previously been shown to include both host-restricted and generalist strains (5, 38).

Conservation of gut symbiont lineages is facilitated by bumblebee eusociality, which allows inter-colony transmission and long-term codiversification between symbionts and hosts (5). It may also reflect the fundamental importance of symbionts to bumblebee biology. For example, microbiome-based parasite protection, a well-documented function in temperate bumblebees (4, 39), is likely important in the tropics. Although the core gut microbiome is generally similar between temperate and neotropical bumblebees, there may be differences that we are unable to detect, such as in strain-level composition and functional potential. The core symbionts of bumblebees generally comprise multiple subspecies and strains that are not differentiated by 16S rRNA gene amplicon sequencing (12, 40). These strains may have different gene content and functional capabilities (41).

Unlike most macroorganismal communities (42), bumblebee gut microbiomes do not exhibit higher alpha diversity in the tropics. Although strain-level analyses are not available, this discrepancy provides evidence that biogeographic patterns based on macroorganisms, such as the latitudinal diversity gradient, do not always apply to microbes (43, 44). Arguably, biogeographic patterns will be even less predictable for specialized host-associated symbionts such as the core gut bacteria of bumblebees, as compared with free-living microbes. Factors such as dispersal, niche availability, and diversification are largely driven by the host in potentially idiosyncratic ways.

Some neotropical bumblebees harbored a highly disrupted microbiome, in which the conserved core gut bacterial symbionts were replaced by environmental microbes similar to those of the solitary bee *Thygater*. This similarity could be due to horizontal transmission via flowers, as *Thygater* and some of the bumblebees were collected foraging in the same area. Notably, disrupted bumblebee microbiomes are highly variable, with phylogenetically and metabolically distinct bacteria (e.g., *Fructobacillus*, *Commensalibacter*, and *Pseudomonas*) present at very different relative abundances in different individuals. Although larger sample sizes are needed for a robust test, our data seem to match theoretical expectations that host-associated communities become more variable with disturbance (45).

Gut microbiome disturbance has also been widely observed in temperate bumblebee species (6, 46) and parallels the findings of discrete microbiome profiles in mammals (42, 47). Thus, not only are the core symbionts conserved in neotropical species, but also their loss and replacement by environmental bacteria. Some of the same bacteria abundant in disturbed gut microbiomes of neotropical bumblebees (e.g., *Fructobacillus*, *Enterobacteriaceae*) are also abundant in disturbed microbiomes of temperate species (43). This overlap may contribute to the clustering of disturbed microbiomes from both regions and is likely due to the widespread occurrence of these taxa in flowers and other substrates in the environment.

The drivers of bumblebee gut microbiome disruption are not yet fully resolved. We found that bumblebee species differed in the prevalence of microbiome disturbance, with the highest prevalence in *B. atratus*. One possible explanation is that *B. atratus* was sampled later in the colony cycle, as microbiome disruption has been linked to colony age (43). The distribution of *Schmidhempelia* tentatively supports this hypothesis. Previous studies have shown that *Schmidhempelia* declines in abundance with individual age in *B. impatiens* (temperate bumblebee) workers (40) and, in this case, is absent in all *B. atratus* samples. Given that old colonies tend to have more old individuals, this pattern may signify that *B. atratus* was sampled later in the colony cycle. Stressors varying across the landscape may also explain interspecific variation in the prevalence of microbiome disruption. All *B. atratus* samples, and the *B. hortulanus* samples with disrupted microbiomes, were collected at lower elevations (Fig. 1). However, many environmental variables are hard to disentangle. For example, among our sample sites in Colombia, low elevations coincide not only with higher temperatures and different plant communities but also with higher levels of anthropogenic disturbance. Indeed, all *B. rubicundus* samples—none of which had disturbed microbiomes—were collected near the *páramos*, defined as protected ecosystems with less direct influence of human activity. For future research, it would be important to meticulously assess such effects. However, in this study, conducting a detailed comparison was unfeasible due to constraints imposed by the number of species and samples.

The microbiome disruption phenomenon has important implications for bee health and, therefore, also for pollination services. Among the neotropical bumblebees, we found a correlation between microbiome disruption and the presence of known bumblebee gut parasites (*Nosema* and *Crithidia*). Studies on wild temperate bumblebees have also linked *Crithidia* infection to variation in gut bacterial community composition (43, 44). Although we cannot distinguish whether parasite infection affects or is affected by gut bacterial disturbance, previous laboratory-based studies support the latter hypothesis (4, 39, 48, 49).

### Conclusions

By interpreting microbial patterns in light of the host’s historical biogeography, we conclude that neotropical bumblebees retained their ancient gut bacterial symbionts during dispersal from North America. Despite the very different environmental conditions and plant communities present in the neotropics, gut microbiome diversity and composition have not strongly diverged from temperate bumblebee microbiomes. Across a broad swath of bumblebee species, microbiomes are more strongly structured by host phylogeny than by geography. However, latitudinal differences in the strain diversity, physiology, and functions of bee gut microbiomes need to be investigated. We also discovered that, similar to temperate species, neotropical bumblebee microbiomes are prone to major disruption. While the cause is unclear, we find an association between the loss of core gut bacteria and parasite infection. This shift may have implications for bumblebee health and pollination services in the neotropics.

## Data Availability

Raw reads are available in the ENA database (accession number: PRJEB42751).

## SUPPLEMENTAL MATERIAL

Fig. S1 msphere.00139-23-s0001.tif ASVs that significantly differ in relative abundance among host species.

Supplemental material legends msphere.00139-23-s0002.docx Legends for Fig. S1 and Tables S1 to S3.

Table S1 msphere.00139-23-s0003.txt Metadata associated with the project.

Table S2 msphere.00139-23-s0004.txt ASV table associated with the project.

Table S3 msphere.00139-23-s0005.csv List of ASVs that significantly differ in relative abundance among host species. ANCOM-BC output.

ASM does not own the copyrights to Supplemental Material that may be linked to, or accessed through, an article. The authors have granted ASM a non-exclusive, world-wide license to publish the Supplemental Material files. Please contact the corresponding author directly for reuse.
