# Supplementary figures and images for: Conserved, yet disruption-prone, gut microbiomes in neotropical bumblebees
Source: mSphere. 2023 Oct 19;8(6):e00139-23. doi: 10.1128/msphere.00139-23 (PMC10732019; doi:10.1128/msphere.00139-23)

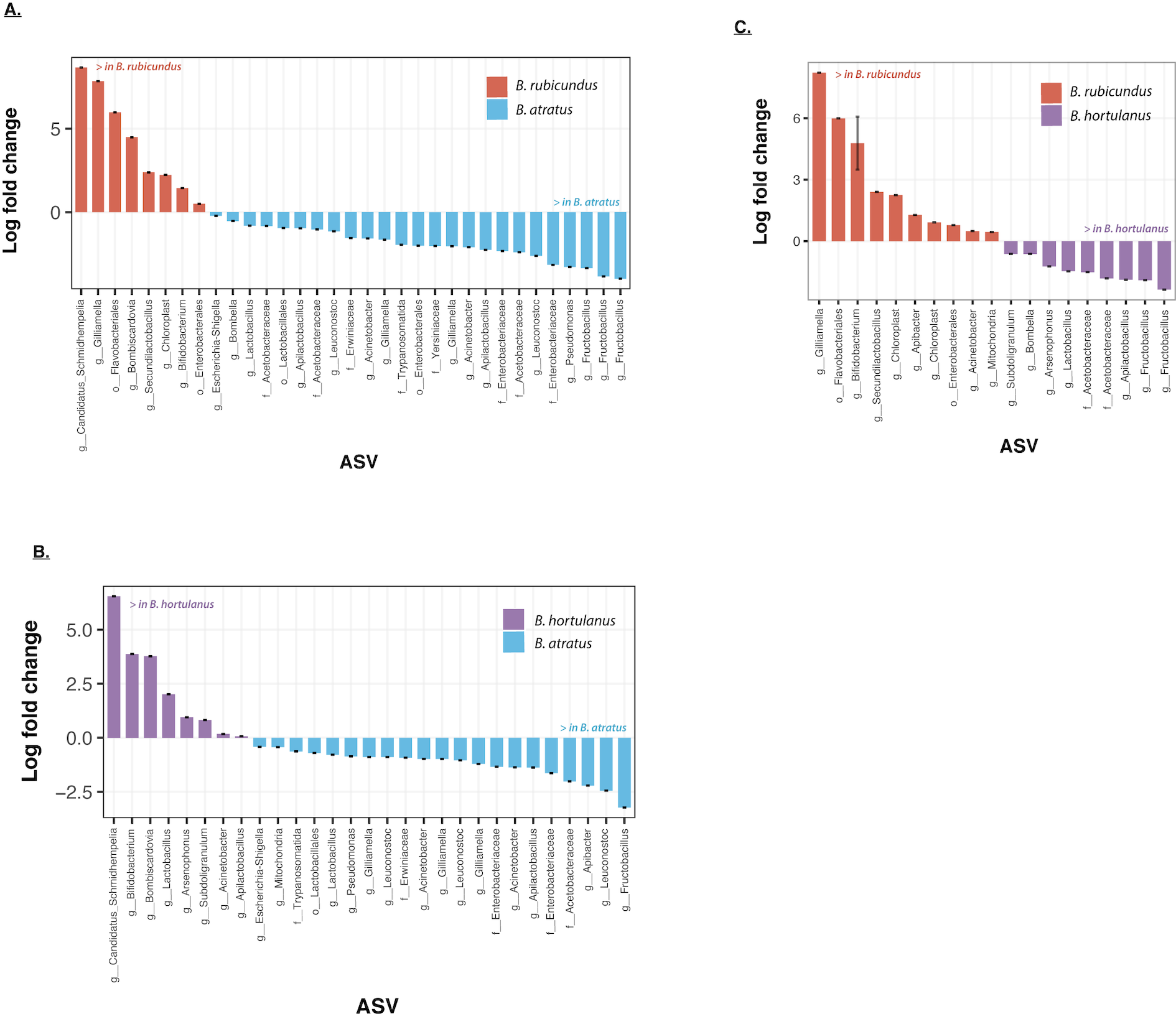

Supplement: Fig. S1 — ASVs that significantly differ in relative abundance among host species. [file msphere.00139-23-s0001.tif]
