# Supplementary material for: Conserved, yet disruption-prone, gut microbiomes in neotropical bumblebees
Source: mSphere. 2023 Oct 19;8(6):e00139-23. doi: 10.1128/msphere.00139-23 (PMC10732019; doi:10.1128/msphere.00139-23)
Supplement: Supplemental material legends — Legends for Fig. S1 and Tables S1 to S3. [file msphere.00139-23-s0002.docx]

**Supplemental material**

**Figure S1.** ASVs that significantly differ in relative abundance among host species.

**Table S1.** Metadata associated with the project.

**Table S2.** ASV table associated with the project.

**Table S3.** List of ASVs that significantly differ in relative abundance among host species. ANCOM-BC output.
